# Surface tension of model tissues during malignant transformation and epithelial–mesenchymal transition

**DOI:** 10.3389/fcell.2022.926322

**Published:** 2022-08-30

**Authors:** Irène Nagle, Alain Richert, Michael Quinteros, Sébastien Janel, Edgar Buysschaert, Nathalie Luciani, Henry Debost, Véronique Thevenet, Claire Wilhelm, Céline Prunier, Frank Lafont, Teresita Padilla-Benavides, Mathieu Boissan, Myriam Reffay

**Affiliations:** ^1^ Laboratoire Matière et Systèmes Complexes, UMR 7057, Université Paris Cité and CNRS, Paris, France; ^2^ Molecular Biology and Biochemistry Department, Wesleyan University, Middletown, CT, United States; ^3^ Univ. Lille, CNRS, Inserm, CHU Lille, Institut Pasteur Lille, U1019—UMR 9017—CIIL—Center for Infection and Immunity of Lille, Lille, France; ^4^ Sorbonne Université, Centre de recherche Saint-Antoine, CRSA, Paris, France; ^5^ Physico-Chimie Curie, Institut Curie, CNRS UMR 168, Paris, France

**Keywords:** mechanobiology, migration, invasion, adhesion, multicellular aggregates, breast tumor, magnetic nanoparticles

## Abstract

Epithelial–mesenchymal transition is associated with migration, invasion, and metastasis. The translation at the tissue scale of these changes has not yet been enlightened while being essential in the understanding of tumor progression. Thus, biophysical tools dedicated to measurements on model tumor systems are needed to reveal the impact of epithelial–mesenchymal transition at the collective cell scale. Herein, using an original biophysical approach based on magnetic nanoparticle insertion inside cells, we formed and flattened multicellular aggregates to explore the consequences of the loss of the metastasis suppressor NME1 on the mechanical properties at the tissue scale. Multicellular spheroids behave as viscoelastic fluids, and their equilibrium shape is driven by surface tension as measured by their deformation upon magnetic field application. In a model of breast tumor cells genetically modified for NME1, we correlated tumor invasion, migration, and adhesion modifications with shape maintenance properties by measuring surface tension and exploring both invasive and migratory potential as well as adhesion characteristics.

## Introduction

In his seminal work, *On growth and form* (Thompson, 1992), D’Arcy Thompson first described the notion that the shapes of biological tissues are determined by physical principles. How shapes emerge from cellular interactions and their physical properties has been a central question in biology for decades. The fact that cells in suspension are observed to be round and that experimental data have shown that mixing cell populations drives cell sorting suggests the existence of a tissue surface tension (Steinberg, 1963). In fluids, both the Young–Laplace law and the Young–Dupré equation describe the shape of droplets and their wetting properties by simple force balance introducing surface tension. By analogy, in tissues, surface tension is essential to determining tissue shape (Ehrig et al., 2019; Hashmi et al., 2022). Considering cells in tissues as molecules in fluids, surface tension at the tissue scale is related to the energy difference between cells in the bulk and cells at the surface of the tissue. This fluid analogy about tissue behavior is still valid under force application: when compressed, a multicellular aggregate flattens due to modification of the cell position with cells pushed to the surface (Hayashi and Carthew, 2004). This increases the actual surface area, dissipating the applied force and minimizing the overall energy (Merkel and Manning, 2017; Hannezo and Heisenberg, 2019). To be predictable, these macroscopic physical properties have to be correlated with more microscopic biological insights. Surface tension formally depends both on the adhesion energy between cells and on the interaction area (Amack and Manning, 2012). Cell–cell adhesion was first identified as a key component of tissue surface tension (Foty and Steinberg, 2005) giving rise to the differential adhesion hypothesis (DAH) postulated by Steinberg. Investigations of the DAH explored *in vitro* actually revealed the predominant role of actin contractility in surface tension (Maître et al., 2012) and shed light on the multitude of factors that contribute to surface tension (Brodland, 2002; Krieg et al., 2008; Heer and Martin, 2017; Nagle et al., 2021). The DAH was, therefore, extended to effective adhesion to take into account both cell–cell adhesion and cell mechanical properties implied in the surface contact area (Manning et al., 2010; Gonzalez-Rodriguez et al., 2012). Thus, surface tension has been hypothesized as being highly sensitive to any modification in cytoskeletal organization and intercellular adhesion.

Cells in living organisms experience physical forces, such as compression, tension, hydrostatic pressure, and shear stress (Northcott et al., 2018). They respond to these forces by modifying their shape and by generating forces. In this regard, tumor cells behave abnormally because they have lost cell–cell adhesion and have growth defects (Pham et al., 2010; Zhang et al., 2020) that give rise to abnormal shapes. Moreover, by remodeling the extracellular matrix, tumor cells can invade adjacent tissue (Mierke, 2020). Tumor progression seems to require abnormal adhesion and mechanical properties at the individual cell scale. How these cell properties translate at the collective tridimensional scale is still unknown. Tridimensional multicellular spheroids appear to be the simplest models to mimic a tissue (Hirschhaeuser et al., 2010), especially in the context of tumors. They provide a potential tool to decipher metastatic potential as long as two criteria are met: 1/obtaining a reproducible perfectly controlled model tissue, and 2/identifying easily measurable macroscopic properties that can serve as a hallmark of metastatic potential. Surface tension as an index of shape generation and maintenance is an appealing candidate indicator of change in mechanical properties and, thus, the invasive potential of tumors. To measure surface tension from model tissues, one has to be able to deform them. Most techniques used to mechanically stimulate spheroids involve confining them either by encapsulation (Alessandri et al., 2013), application of osmotic pressure (Montel et al., 2011), or compression between rigid plates. Magnetic compression, by contrast, utilizes magnetic nanoparticles (Mazuel et al., 2015) to exert volume forces on the cells, mimicking the stress experienced by tumors due to extracellular matrix stiffening and abnormal tissue growth (Mary et al., 2022).

Epithelial–mesenchymal transition (EMT) is often associated with metastasis. The transition from an epithelial to a mesenchymal phenotype is not a simple switch; it comprises a large spectrum of phenotypes resulting in decreased cell–cell adhesion and enhanced migration and invasion (Pastushenko and Blanpain, 2019). However, most studies on the biomechanics of tumor cells focus on malignant transformation and do not consider EMT. NME1, first identified as a metastasis suppressor (Steeg et al., 1988; Boissan et al., 2005), is an inhibitor of EMT (Huna et al., 2021). Its loss induces a hybrid state of EMT intermediate between fully epithelial and fully mesenchymal states, that represents an unprecedented way to look at biophysical tool sensitivity (Huna et al., 2021). While there are now around 30 identified metastasis suppressor genes (Khan and Steeg, 2018), NME1 was first discovered and the most extensively characterized at the mechanistic and clinical levels. Its expression in melanomas and in epithelial tumors such as breast, liver, colon, and cervical carcinomas shows an inverse correlation with metastatic potential. This inverse relationship between NME1 expression and metastatic potential is most strongly observed in breast tumors. NME2 is a closely related isoform of NME1. While the two proteins are 88% identical in sequence and share many common properties, the role of NME2 is far from being elucidated.

In this study, we investigate how EMT affects surface tension by inducing the loss of NME1 and one of its close isoforms, NME2, in a purely cellular tridimensional model tissue. We also explore the relationship between surface tension and adhesion in accordance with the differential adhesion hypothesis upon NME1 or NME2 inactivation. Moreover, we explore the role of both NME1 and NME2 in such metastasis-associated biological processes as EMT, migration, and invasion by using a breast tumor cell line model. We also shed light on the relationship between surface tension and more dynamic parameters such as migration and invasion, which are major hallmarks of EMT upon NME1 or NME2 inactivation. We show that surface tension decreases not only during the transition from a normal to a malignant cellular state but also during tumor progression across EMT. Thus, reduction of surface tension can be used as a readout of malignant transformation and tumor aggressiveness.

## Materials and methods

### CRISPR/Cas9 gene editing

#### CRISPR guides

Lentiviral plasmid guides targeting human NME1 and NME2 were generated in the pLenti U6gRNA Cas9-GFP-Puro vector. These vectors and the non-target guide (pLenti CRISPR-NT CONTROL) were purchased from Merck-Sigma-Aldrich. Two different guides were designed for both NME1 and NME2: NME1(#A) (*#*HS0000009943, target sequence GAC​GGG​CCG​AGT​CAT​GCT​CGG​G), NME1(#B) (*#*HS0000009940, target sequence GAA​CAC​TAC​GTT​GAC​CTG​AAG​G), NME2(#A) (*#*HS0000056847, target sequence TCA​TCG​CCA​TCA​AGC​CGG​ACG​G), and NME2(#B) (*#*NME2-0-76, target sequence AAG​ACC​GAC​CAT​TCT​TCC​CTG​G).

#### Lentiviral vectors productions and MCF10DCIS.com cells transduction

These steps were performed with the help of the GIGA Viral vectors platform (University of Liège, Belgium). In brief, Lenti-X 293T cells (Clontech) were co-transfected with pcgpV (Cell Biolabs), pRSV-Rev (Cell Biolabs), and VSV-G (Cell Biolabs) encoding vectors together with pLenti U6gRNA NME1-Cas9-GFP-Puro or pLenti U6gRNA NME2-Cas9-GFP-Puro or pLenti CRISPR-NT CONTROL. Lentiviral supernatants were collected 48–96 h post-transfection, filtrated, and concentrated 100x by ultracentrifugation. Lentivirus stocks were titrated with qPCR Lentivirus Titration (Titer) Kit (abm) and used to transduce cells. After 72 h, cells were selected with 2 μg/ml puromycin (Cayla/Invivogen). Then, cells expressing GFP were isolated and cloned by FACS on a FACSaria III 4L sorter (BD Biosciences). Each clone was tested by Western blotting. Clones that were negative for NME1 or NME2 expression were selected for further experiments.

#### Sequencing

Selected clones were analyzed by miSeq in order to confirm mutations in NME1 or NME2-coding sequences, as previously described (Huna et al., 2021).

### Cell lines and culture

MCF10A cells were obtained from ATCC (CRL-10317) and cultured in DMEM/F12 medium (Gibco) supplemented with 5% horse serum, 20 ng/ml EGF (Sigma-Aldrich), 0.5 μg/ml hydrocortisone (Sigma-Aldrich), 10 μg/ml insulin (Sigma-Aldrich), 50 μg/ml Bovine Pituitary Extract (Gibco). MCF10DCIS.com cell line was purchased from Asterand. MCF10DCIS.com cells invalidated for NME1 or NME2 were obtained by CRISPR-Cas9 gene editing, as described in the previous section. The MCF10DCIS.com cells and their derivatives were cultured in an advanced DMEM/F12 medium supplemented with 5% horse serum and 2 mM glutamine. All cells were maintained at 37°C in a 5% CO_2_ atmosphere.

### Proteins extraction and Western blotting

Proteins from cell extracts were electrophoretically separated on 10% sodium dodecyl sulfate (SDS) polyacrylamide gels, transferred onto nitrocellulose membranes, and probed with highly specific NME1 and NME2 rabbit polyclonal antibodies (Boissan et al., 2005). Immunoblots were revealed with peroxidase-coupled secondary antibodies and enhanced chemiluminescence (ECL) Plus substrate (GE Healthcare). *α*-tubulin antibodies (Thermo Fisher Scientific) were immunoprobed as indexes of the cellular protein level.

After a rinse with PBS, multicellular spheroids were homogenized and solubilized in ice-cold 30 mM Tris-EDTA, pH 7.2, containing 1 mM DTT, 1% (v/v) Triton X-100, 10% (w/v) anti-phosphatase cocktail and 14% (w/v) anti-protease cocktail (Roche), for 30 min on ice, followed by centrifugation at 12000 g for 20 min at 4°C. The proteins in the supernatants were then quantified using a Bradford assay and used for Western blot analysis. Proteins from multicellular spheroids extracts were separated on 7.5% SDS–polyacrylamide gels and electroblotted onto PVDF membranes. After being rinsed in TBS-Tween 20 buffer (TBST), the blots were blocked for 1 h in TBST with 5% (w/v) non-fat dry milk, then probed overnight at 4°C with either E-cadherin (1:1,000, Cell Signaling *#*14472) or N-cadherin (1:1,000, Sigma-Aldrich, *#*SAB5700641) specific antibodies. After three washes with TBST, the blots were incubated with horseradish peroxidase-linked anti-rabbit Ig from sheep. Peroxidase activity was revealed with a chemiluminescent detection kit (ECL Plus substrate, GE Healthcare), Beta-actin antibodies (Thermo Fisher Scientific) were immunoprobed as indexes of the cellular protein level and analysis was processed by ImageJ software.

### Atomic force microscopy

Atomic force microscopy (AFM) experiments were performed on a JPK NanoWizard^®^ III system (Bruker, Berlin, Germany) coupled to a Zeiss Axio Observer. Z1 optical microscope with a ×40 air objective mounted on a PIFOC (Physik Instrumente, Karlsruhe, Germany). The AFM was equipped with an additional Z piezo scanner of 100 μm (JPK CellHesion) and the FluidFM^®^ technology add-on (Cytosurge, Glattbrugg, Switzerland). FluidFM^®^ micropipettes of 4 μm aperture and 0.3 N/m nominal spring constant were used. Micropipettes were first cleaned in plasma oxygen for 2 min at 20W. They were then covered with 0.1 mg/ml PLL (20)-g [3.5]-PEG (2) (SuSoS, Dübendorf, Switzerland) both inside and outside the cantilever for 1 h in order to ease cell release. Micropipettes were then rinsed in ultrapure water and mounted on the dedicated holder. The real spring constant was determined using the off-contact Sader method after 5 min thermalization.

**FIGURE 1:**
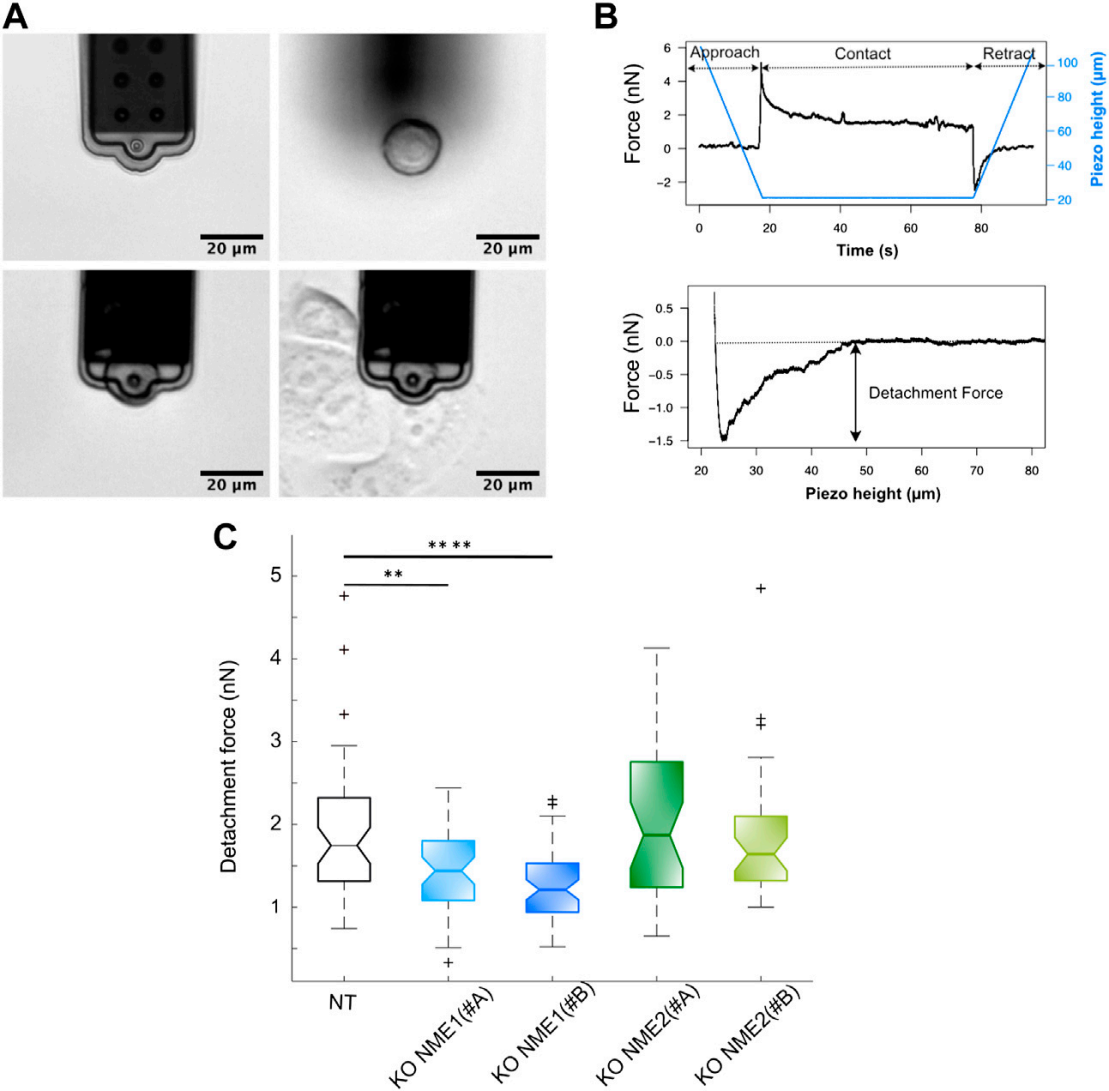
Inactivation of NME1 reduces cell–cell adhesion force. **(A)** Top left: FluidFM^®^ micropipette. Top right: suspension cell sedimented on the dish. Bottom left: cell picked up by negative pressure in the micropipette channel. Bottom right: cell vs. cell contact. **(B)** Top: piezo height vs. time (blue) and force vs. time (black) plots of a cell vs. cell interaction. Force increases up to 5 nN for contact, and then height is kept constant for 60 s as cells relax. Finally, cantilever is retracted at a constant speed, and a negative detachment force is observed. Bottom: force vs. piezo height plot example where detachment force is measured. **(C)** Boxplots of detachment forces from MCF10DCIS.com cells in which NME1 or NME2 was inactivated (on average, *n* = 45 cells were tested per condition). Notch plots show means ± SEM and first to third quartiles of three independent biological replicates measured. ****p < 0.0001; **p < 0.01 relative to NT control cells.

Cells were cultured on several 40 mm Petri dishes (TPP, Trasadingen, Switzerland) in a complete medium. Suspension cells were obtained by incubation in 0.05% trypsin-EDTA for 5 min at 37°C to detach the cells, followed by trypsin inactivation in a complete medium for 30 min at 37°C in a CO_2_ incubator. Another dish of cells was cultured in an advanced DMEM/F12 medium complemented with 10 mM HEPES buffer (imaging medium) before placement on the AFM stage with 37°C temperature control. A few microliters of suspended cells were added to the dish, and the cells were allowed to settle on the bottom of the dish for 1−2 min before being picked up by the micropipette using a soft contact (1 nN) and pressure of −100 mbar. Once picked up, the pressure was decreased to − 10 mbar and the cell was allowed to rest away from the surface for 5 min. This cell was then brought into contact with a spread cell with an initial 5 nN contact force (Figure 1A). The AFM height was kept constant for 60 s, and then the micropipette was retracted at constant velocity (5 μm/s). This same cell was allowed to rest for a few minutes and then brought into contact with three different cells before being released by applying a pressure of 500 mbar. An average number of 45 cell–cell detachment curves with at least three different cell cultures per condition were recorded. The detachment force was analyzed using JPK DP software (6.3.50) as the lowest point in the retraction curve after a baseline correction (Figure 1B).

### Wound healing assay

Cells were grown to confluency on 24-well plates and starved for 24 h in a serum-free medium. They were treated for 2 h with 10 μM Cytosine*β*-D-Arabinofuranoside (AraC) to inhibit cell proliferation. After starvation, cells were scratch-wounded using a sterile 200 μL pipette tip, and suspended cells were removed by washing with PBS twice. Cell migration into the wound was monitored every 24 h until wound closure using the ×10 objective of an Echo Rebel microscope. The bottom of the plate was marked for reference, and the same field of the monolayers was photographed immediately after performing the wound (*t* = 0 h) and at different time points after the scratch. A total of 30 scratches were analyzed for each condition from three independent experiments.

### Matrigel invasion assay

Matrigel invasion assay was performed following the Transwell chamber method as described (Olea-Flores et al., 2019). In brief, BioCoat Matrigel invasion chambers with 8.0 μm PET membranes placed in 6-well plates were used to seed cells that were previously treated for 2 h with 10 μM Cytosine *β*-D-Arabinofuranoside (AraC) to inhibit cell proliferation. The cells were plated at a density of 1.25 × 10^5^ cells/mL in 2 ml of a serum-free medium on the top chamber, as recommended by the manufacturer. The lower chamber of the Transwell contained 2.5 ml of culture medium supplemented with serum. Cells were incubated for 24 h at 37°C in a 5% CO_2_ atmosphere. Following incubation, cells and Matrigel on the upper surface of the Transwell membrane were gently removed with cotton swabs. Invading cells on the lower surface of the membrane were washed and fixed with methanol for 5 min and stained with 0.1% crystal violet diluted in PBS. Images from 5 fields of three independent biological replicates were taken and used for cell quantification using FIJI software, version 1.44p (NIH, Bethesda, MD, USA) (Schindelin et al., 2012).

### Magnetic cell labeling

Iron oxide superparamagnetic nanoparticles (8 nm diameter) were obtained by alkaline coprecipitation, followed by oxidation into maghemite according to the Massart procedure (Boitard et al., 2021). The aqueous solution was stabilized electrostatically by adsorbing citrate anions to the surface of the nanoparticles.

MCF10A cells were incubated for 23 min with a solution of iron oxide nanoparticles with [*Fe*] = 1 mM supplemented with 5 mM citrate in RPMI medium (Gibco), while the other cell types were incubated for 45 min in a solution of iron oxide nanoparticles at [*Fe*] = 4 mM and supplemented with 5 mM citrate in RPMI medium. The labeling medium was then discarded and replaced by a complete medium for at least 2 h before cells were trypsinized and detached. Cell proliferation after magnetic labeling was assessed using Alamar Blue (Sigma-Aldrich) assay.

### Magnetic molding

Labeled cells were seeded in semi-spherical 2% agarose molds due to magnet attraction. Agarose molds were obtained from agarose gelification around 1.2 mm steel beads (BI 00151, CIMAP) as previously described (Mazuel et al., 2015). Spheroids were incubated overnight at 37°C, 5 %CO_2_ in a complete medium and extracted from the wells by gently pipetting the surrounding liquid. The resulting spheroids had a radius of 450 ± 70 μm.

### Magnetic force tensiometer

Magnetic forces were exerted by a 6 × 6 mm cylindrical neodymium permanent magnet (S-06-06-N, Supermagnete). The generated magnetic field is almost constant over the aggregate height at around 530 mT, and the magnetic gradient [grad(B)] was 170 T/m. One multicellular aggregate was deposited at 37°C in a temperature-regulated tank, whose bottom interface is made of a non-adhesive treated glass slide (30 min incubation with anti-adherence rinsing solution from Stemcell Technologies). Images of the aggregate profile were taken using a FLIRFly camera (Teledyne FLIR) equipped with a 1.5× zoom lens and an additive 5 × lens (Thorlabs) through sealed glass slides. The magnet is approached at 150 μm from the bottom of the multicellular spheroid. The equilibrium shape of the multicellular aggregate is reached after 10 min. The surface tension *γ* is deduced from the flattened profile of the aggregate by using the TensioX dedicated MatLab application (Nagle et al., 2021). In brief, it integrates Laplace laws for capillarity and minimizes the quadratic error on the height (*h*), width (*w*) and volume (*V*) of the spheroid (Kalantarian et al., 2015) to extract the capillary constant $c=\frac{M_{V}\mathrm{grad}\left(B\right)}{\gamma }$ , where *M*_*V*_ represents the magnetic moment per unit volume. Indeed, the profile can be described by the following equation derived from Laplace laws (Kalantarian et al., 2015): $\frac{d\phi }{ds}=2b+cz-\frac{\sin\left(\phi \right)}{x}$ , where *ϕ* is the angle of inclination of the profile, *s* stands for the arc length along the profile, and *b* is the curvature at the apex. By assessing *M*_*V*_ with vibrating sample magnetometry (VSM) measurements, the surface tension *γ* can be deduced.

### Indirect immunofluorescence analysis

Cells in 2D monolayers grown on glass coverslips were fixed with 4% paraformaldehyde for 15 min, permeabilized with 0.1% Triton X-100 for 5 min, and then incubated with either anti-E-cadherin rabbit monoclonal antibody (1:200; Cell Signaling Technology Inc.) or anti-Pan-cadherin rabbit polyclonal antibodies (1:100; Sigma-Aldrich). The secondary antibody used was AlexaFluor 488-conjugated goat anti-rabbit IgG (ThermoFisher Scientific). Nuclei were stained with DAPI (ThermoFisher Scientific). Images were obtained by confocal microscopy (Leica equipped with a ×40 water immersion objective).

Multicellular spheroids were fixed with 4% paraformaldehyde for 60 min permeabilized with 1% Triton X-100 for 2 days. They were incubated for 24 h with E-cadherin rabbit polyclonal antibodies (1:200; Cell Signaling Technology Inc.) at 4°*C*. The secondary antibody used was AlexaFluor 488-conjugated goat anti-rabbit IgG (ThermoFisher Scientific). Nuclei were stained with Hoechst 3342 (Invitrogen). Images were obtained by confocal microscopy (Zeiss LSM780 with a ×20 water immersion objective).

### Alamar blue metabolic assay

The metabolic activity of the cells was quantified using the Alamar Blue assay. For nanoparticles condition, the assay was performed 2 h and 1 day after the magnetic cell labeling while control cells were not labeled. The Alamar Blue reagent (Sigma-Aldrich) was incubated (10% in DMEM) with each cell type for 1 h according to supplier protocol, and the signal was detected using a fluorescence plate reader (Enspire, Perkin Elmer) at 570 nm excitation wavelength and 585 nm emission wavelength in 96-well plates.

### Statistics

Graphs and statistics were processed using MatLab. Notched boxplots were used to represent the median, the first and third quartiles and the confidence interval for comparison. All statistical tests were performed with a two-sided Mann–Whitney U test (Wilcoxon test) using MatLab. *p*-value is used to indicate the statistical significance of the results: *, **, ***, ****, ***** correspond to *p* < 0.05, *p* < 0.01, *p* < 0.001, *p* < 0.0001, and *p* < 0.00001, respectively.

## Results

### Characterization of EMT-associated biological processes driven by inactivation of NME1

Cells undergoing EMT lose epithelial characteristics and integrity, acquire mesenchymal features, and become further motile and invasive (Brabletz et al., 2021). Thus, decreased cell–cell adhesion and enhanced migration and invasion are considered hallmarks of EMT.

To obtain cell lines in which the NME1 or NME2 genes were completely and stably inactivated, we performed CRISPR–Cas9 gene editing in the human breast tumor cell line MCF10DCIS.com using two independent guide RNAs specific to the NME1 gene, NME1 (#A) and NME1 (#B), and two for the NME2 gene, NME2 (#A) and NME2 (#B). To provide a control cell line for experiments with the NME1-and NME2-ablated cells, we subjected MCF10DCIS.com cells to the CRISPR–Cas9 procedure but omitted a guide RNA [No-targeting (NT) cells] (Huna et al., 2021). The ablation of the two proteins, NME1 and NME2, was validated by immunoblot analysis in the different clones (Supplementary Figure S1). NME1-ablated cells, unlike NME2-ablated cells, are the only cell line that lose epithelial features and acquire mesenchymal characters (Huna et al., 2021).

#### Specific loss of NME1 reduces cell–cell adhesion force

AFM measurements were performed to explore modifications in cell–cell adhesion force by measuring cell–cell detachment after a short-term adhesion of 60 s. The inactivation of NME1, but not of its closely related isoform NME2, moderately but significantly decreases detachment force. The measured mean adhesion force for NME1-ablated cells is 1.4 ± 0.5 (mean ± std) using the NME1 (#A) guide and 1.2 ± 0.4 nN using the NME1 (#B) guide. The measured mean adhesion force for NME2-ablated cells is 2.0 ± 1 nN using the NME2 (#A) guide and 1.9 ± 0.8 nN using the NME2 (#B). The measured mean adhesion force for NT control cells is 1.8 ± 0.8 nN (Figure 1). Thus, inactivation of NME1 but not of NME2 reduces cell–cell adhesion force.

#### Specific inactivation of NME1 increases directional migration

**FIGURE 2:**
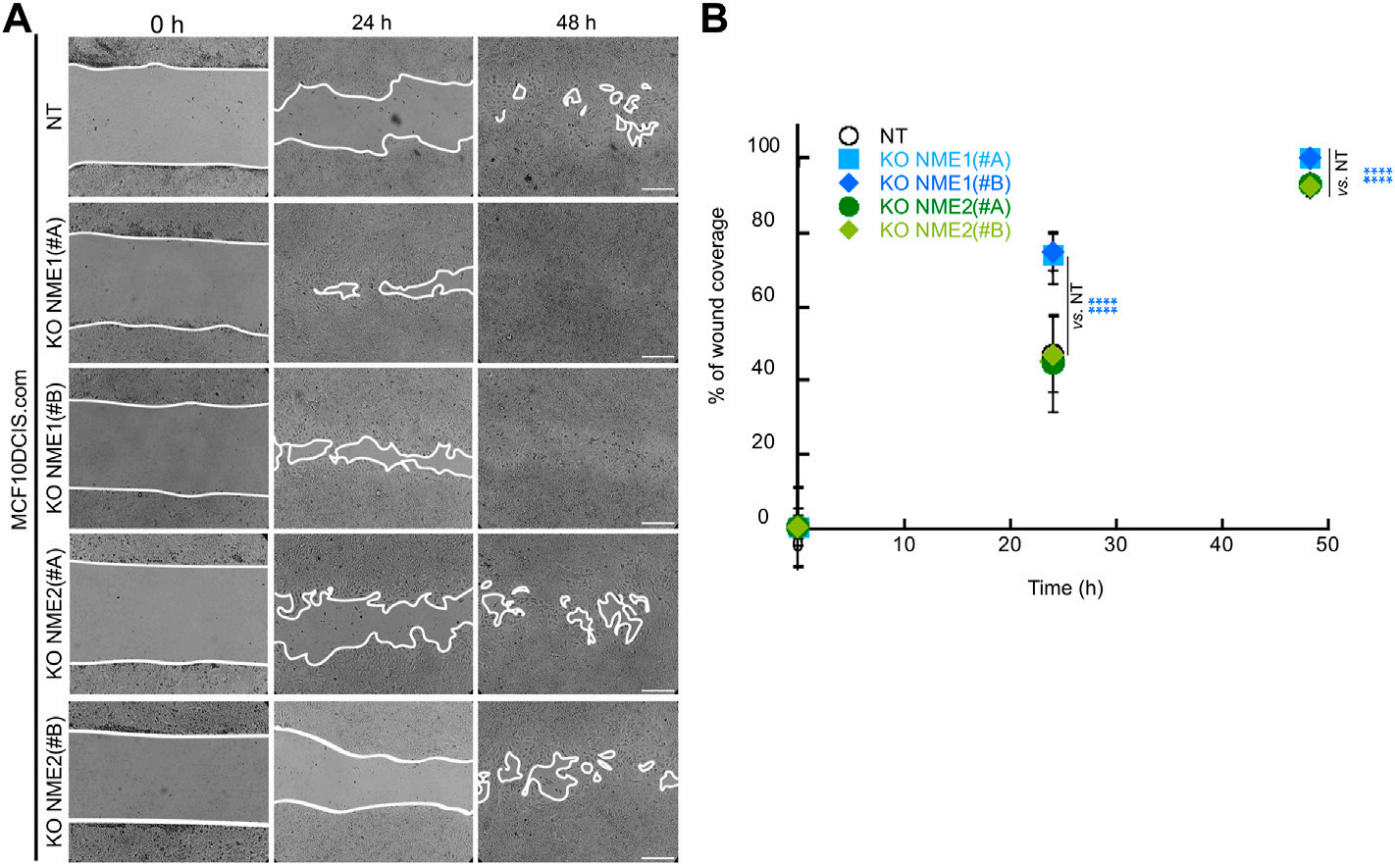
Inactivation of NME1 promotes directional migration. **(A)** Representative light microscopy images of the wound healing assay of MCF10DCIS.com cells in which NME1 or NME2 was inactivated. Time 0 represents confluent monolayer wounds at 0 h, and wounds were monitored until NME1-ablated cell monolayers became fully closed 48 h after scratching the monolayer. Images are representative of three independent biological replicates. Scale bar: 100 μm. **(B)** Quantification of the area of migration over time shown in **(A)**. Data show means ± SD of three independent biological replicates imaged. ****p < 0.0001 relative to NT control cells.

In order to assess the migration of NME1-and NME2-ablated cells, we performed a wound-healing assay, in which a confluent cell monolayer was breached and the degree of migration to close the wound in a given time period was determined. When comparing the wounds immediately after the scratch (0 h) and 24 h later, NME1-ablated cells treated with either guide covered over 75% of the scratched area, whereas the NT control and NME2-ablated cells only covered 55% of the area (Figure 2). After 48 h, NME1-ablated cells had fully covered the wound, whereas NT control and NME2-ablated cells still had open wounded areas that accounted for approximately 10% of the original scratched area (Figure 2). Thus, directional migration induced by wound closure is increased when NME1 is inactivated but not when NME2 is inactivated.

#### Specific inactivation of NME1 increases invasion into the basement membrane

**FIGURE 3:**
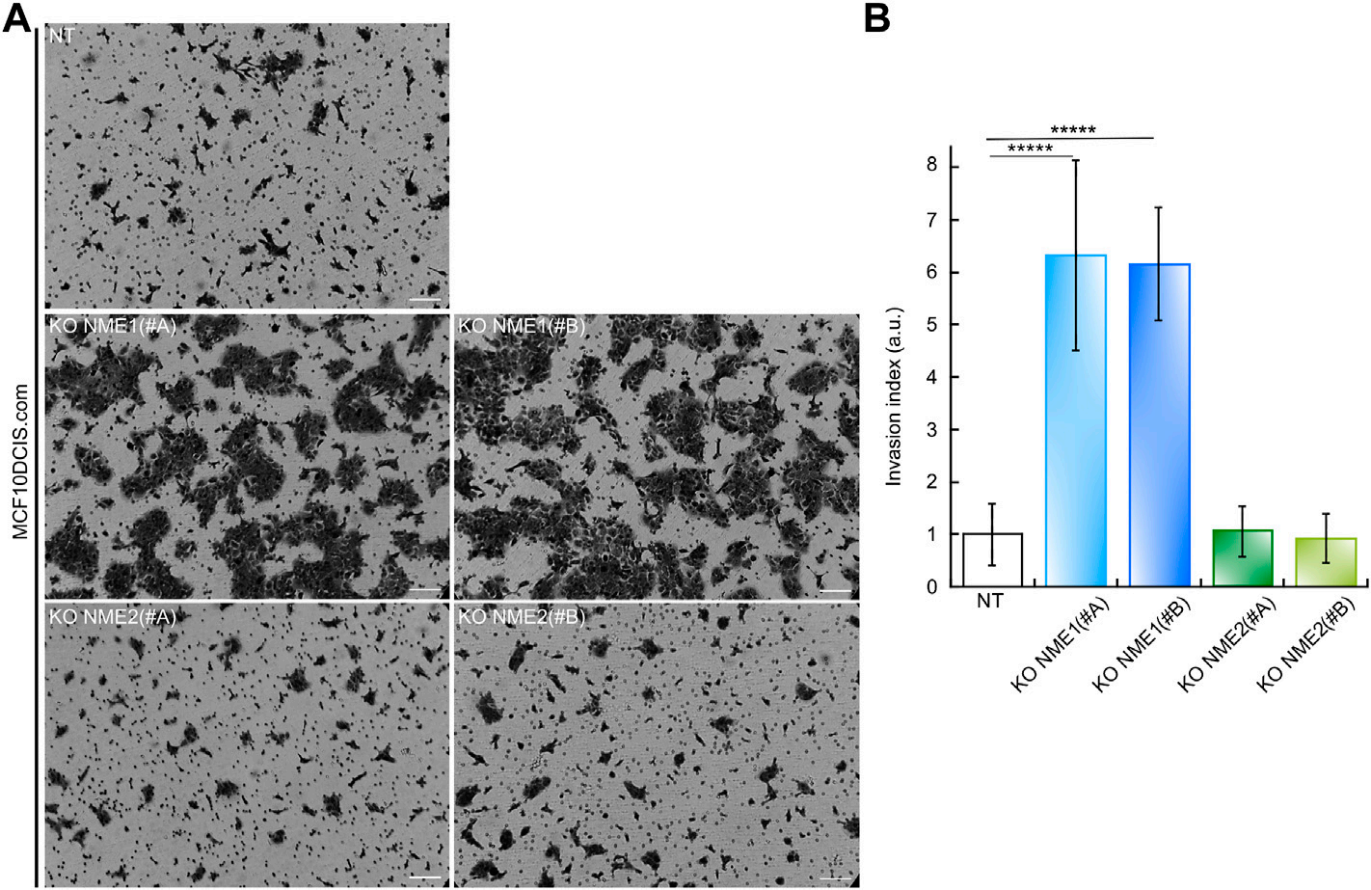
Inactivation of NME1 promotes invasion through Matrigel. **(A)** Representative light microscopy images of Matrigel invasion assay of MCF10DCIS.com cells in which NME1 or NME2 was inactivated after 24 h. (Scale bar: 100 μm) **(B)** Quantitative analysis of the invasion assay presented in **(A)**. The data show the means ± SEM of three independent biological replicates imaged and are expressed as an invasion index defined as the ratio of the number of invading NME1-ablated cells or invading NME2-ablated cells on the number of invading NT control cells. By definition, the invasion index of NT control cells is 1. *****p < 0.00001 relative to NT control cells.

To investigate the functional consequences of inactivating NME1 and NME2 in MCF10DCIS.com cells, we also studied their invasion of Matrigel, a basement membrane extract. Cells were plated on top of a polycarbonate membrane covered with Matrigel, through which invasive cells could cross and invade the opposite side of the membrane (Figure 3). After 24 h of culture, the number of NME1-ablated cells crossing the Matrigel was, on average, much higher than the number of invading NT control cells. This was true whatever the NME1 (#A) or NME1 (#B) guide (Figure 3). Inactivation of NME1 induces a 6.5-fold increase in the invasion index as defined as the ratio of the number of invading cells to the number of invading NT control cells. By contrast, the number of NME2-ablated cells crossing the Matrigel is similar to the number of invading NT control cells for both cells treated with the NME2 (#A) or the NME2 (#B) guide. These data indicate that invasion through Matrigel is increased when NME1 is inactivated but not when NME2 is inactivated.

### Multicellular stimulable spheroids: From two-dimensional to three-dimensional environment

Model tissues of defined cell type, shape and size were obtained by magnetic molding techniques to assess their 3D organization. Using superparamagnetic nanoparticles incorporated into cells through the endocytosis pathway, the cells were given magnetic properties that allowed them to behave like induced magnets that can be either driven or stimulated at will using external magnets. Viability and lack of cytotoxicity of the magnetic labeling were confirmed using the Alamar Blue assay by measuring the unchanged metabolic activity of the cells after labeling (Supplementary Figure S2). Moreover, the incorporation of magnetic nanoparticles was shown to have no impact on cell–cell adhesion, migration, or invasion (Supplementary Figure S3). These magnetic forces can concentrate seeded cells in a well of determined size within 1 min. After 20 h of maturation, a cohesive, perfectly reproducible multicellular spheroid about 1 mm diameter is formed due to cell–cell adhesions.

**FIGURE 4:**
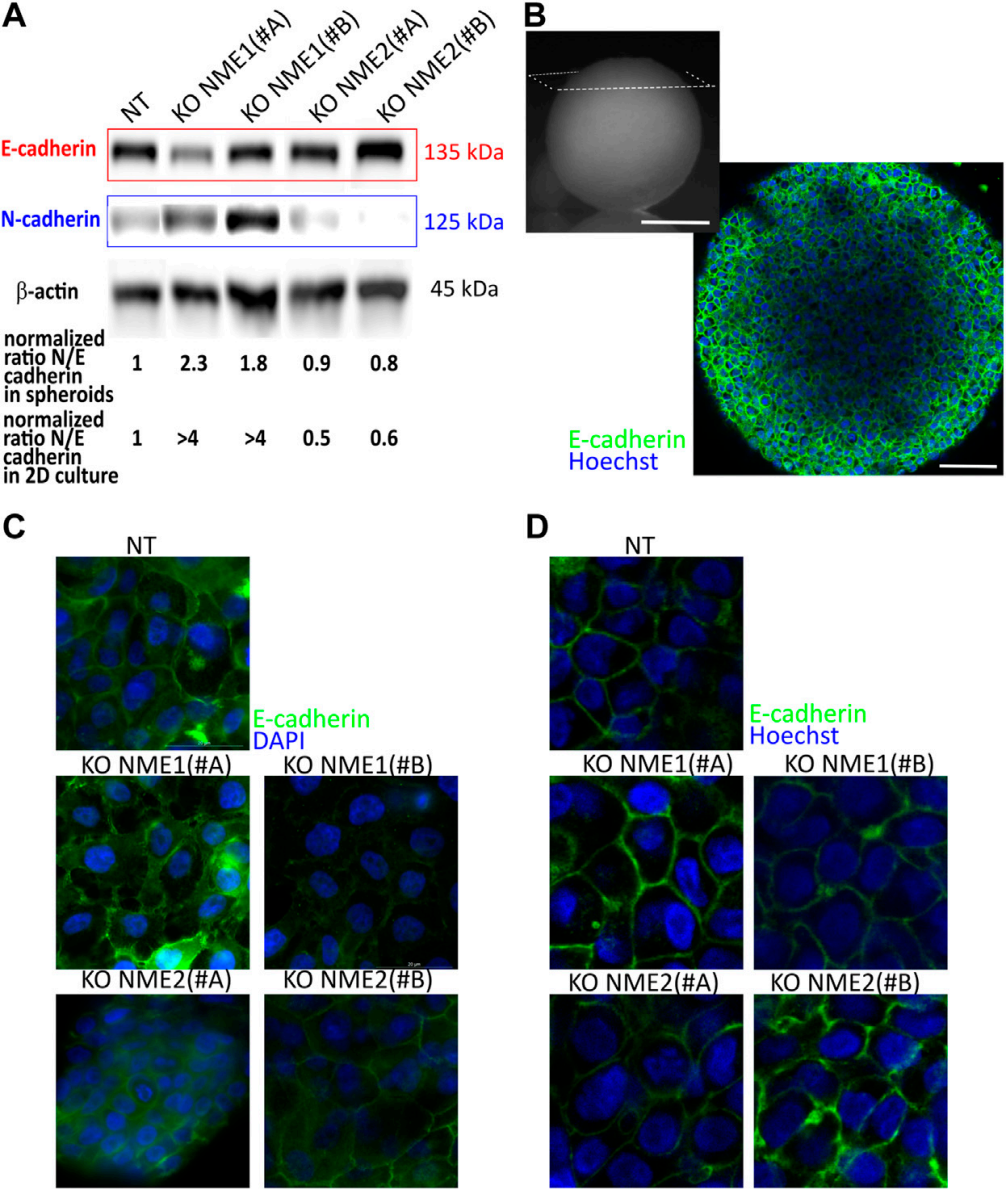
E-cadherin and N-cadherin expression are modified in spheroids of NME1-ablated cells. **(A)** Western blot analysis of E-cadherin and N-cadherin expression in NT-control cells compared to cells where NME1 or NME2 were inactivated. β-actin was used as a loading control. The ratio of N- to E-cadherin is calculated and renormalized to the ratio obtained for NT control cells. This ratio is explored in 3D spheroids and in 2D culture (data analyzed from Huna et al. (2021)). **(B)** Top left: image of a spheroid of 1 mm diameter of NT control cells. The observation plane is indicated in white (Scale bar: 500 μm). Bottom right: image obtained at 70 μm depth penetration of E-cadherin localization. Nuclei are stained by Hoechst (Scale bar: 50 μm). **(C)** Immunofluorescence confocal images of 2D culture cells from MCF10DCIS.com cells in which NME1 or NME2 was inactivated. E-cadherin antibody labeling is used. Nuclei are labeled with DAPI. The field of view is a square of 50 μm-long. **(D)** Immunofluorescence confocal images of multicellular spheroids from MCF10DCIS.com cells in which NME1 or NME2 was inactivated taken at a distance from the top of the aggregate between 50 and 80 μm. The field of view is a square of 50 μm-long. E-cadherin antibody labeling is used. Images of nuclei labeled with Hoechst are superimposed.

We first looked at the organization of the spheroids to see how the modification of cell–cell adhesion is transduced at the tridimensional level. In adherent cells, epithelial integrity is disrupted upon inactivation of NME1 (Figure 4C). By contrast, in tridimensional multicellular spheroids, NME1 and NME2 inactivation do not impact E-cadherin localization (Figures 4B,D). Looking at protein expression, E-cadherin levels are slightly decreased by NME1 inactivation, while N-cadherin expression is increased. These results correspond to the ones observed in 2D culture (Huna et al., 2021), though the impact of NME1 inactivation is reduced in the multicellular spheroid model compared to that in 2D culture (Figure 4A). E-cadherin expression is reduced by approximately 20% compared to the NT-controls, while it was reduced by 40% in 2D culture (Figure 4A). Looking at the N/E cadherin ratio, which is one hallmark of EMT, multicellular spheroids reproduce the tendency observed in 2D cultures meaning that the NME1 inactivation induced an increase of this ratio compared to NT-controls but while the increase is in the 2-fold range in 3D multicellular aggregates, it was noticed in the 4-fold range for 2D culture. As expected, the overall level of total cadherin is also decreased by NME1 inactivation (Supplementary Figure S4). Thus, a tridimensional environment modifies the cadherin expression compared to two-dimensional models.

### Surface tension variations across EMT

#### Specific inactivation of NME1 strongly decreases surface tension

**FIGURE 5:**
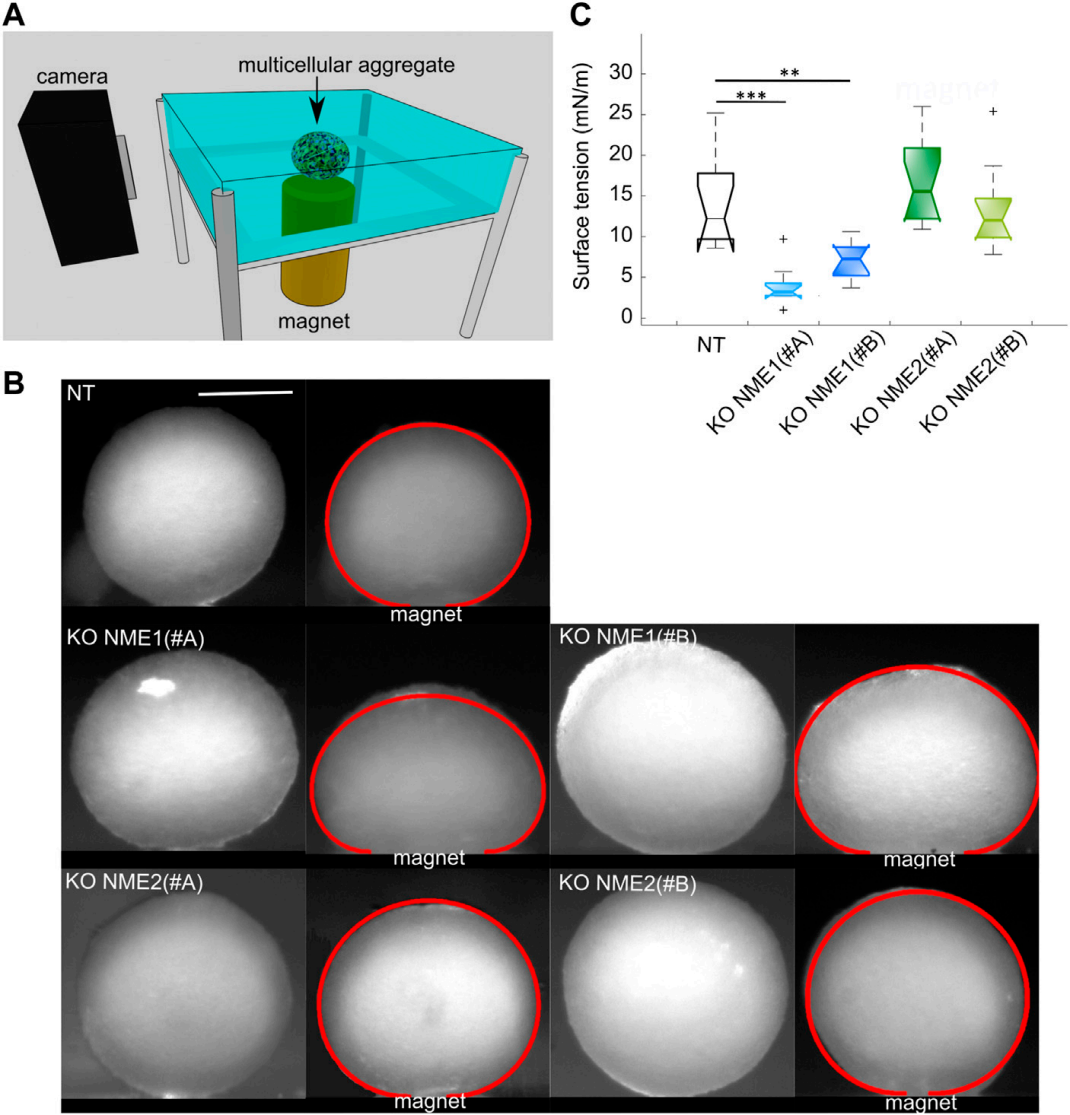
Surface tension is reduced after NME1 inactivation. **(A)** Magnetic tensiometer. A multicellular aggregate is seeded in a glass-side temperature-regulated homemade tank. A camera records its profile from the moment a permanent magnet is put in contact with the bottom slide of the tank. **(B)** Representative side profile images before and after the magnet approach for multicellular aggregates made from MCF10DCIS.com cells in which NME1 or NME2 was inactivated (scale bar: 500 μm). Initial aggregates radii are of a comparable range. The obtained fits of the flattened profiles are superimposed in red. Aggregates of NME1-ablated cells are more flattened than aggregates of either NT or NME2-ablated cells. **(C)** Surface tension obtained from the different cell types presented in **(B)**. Median, standard deviation, and 95% interval of confidence are indicated. Each cell type’s measurements have been repeated over *N* = 3 independent experiments and over 9–15 multicellular aggregates. Only significant tests are indicated (**p < 0.01, ***p < 0.001). Results are compared to NT control cells.

The surface tension of model tissues can be determined by flattening magnetic spheroids *via* a permanent magnet approach (Figure 5A). For a given initial size, the flatter the spheroid looks at equilibrium, the smaller its surface tension is (Mazuel et al., 2015). Surface tension is deduced from the fit of the aggregate profile (David et al., 2009; Kalantarian et al., 2015) (Figure 5B).

Starting from the same spheroid radius, aggregates of NME1-ablated cells, whether treated with the NME1 (#A) or NME1 (#B) guide, look flatter than both the aggregates of NT control and NME2-ablated cells, whether treated with the NME2 (#A) or NME2 (#B) guide (Figure 5B). The surface tension values of spheroids of NME1-ablated cells are 4 ± 3 mN/m (mean ± sd) for cells treated with the NME1 (#A) guide and 7 ± 2 mN/m for those treated with the NME1 (#B) guide. The NT control cell aggregate surface tension value is 16 ± 9 mN/m. By contrast, aggregates of NME2-ablated cells have a surface tension value of 16 ± 5 mN/m and 13 ± 5 mN/m for cells treated with the NME2 (#A) guide and the NME2 (#B) guide, respectively, which is close to the value obtained for the NT control cell aggregates (Figure 5C).

Surface tension is thus strongly modified upon NME1 inactivation, while the inactivation of NME2 does not significantly impact it.

### Surface tension decreases during malignant transformation

**FIGURE 6:**
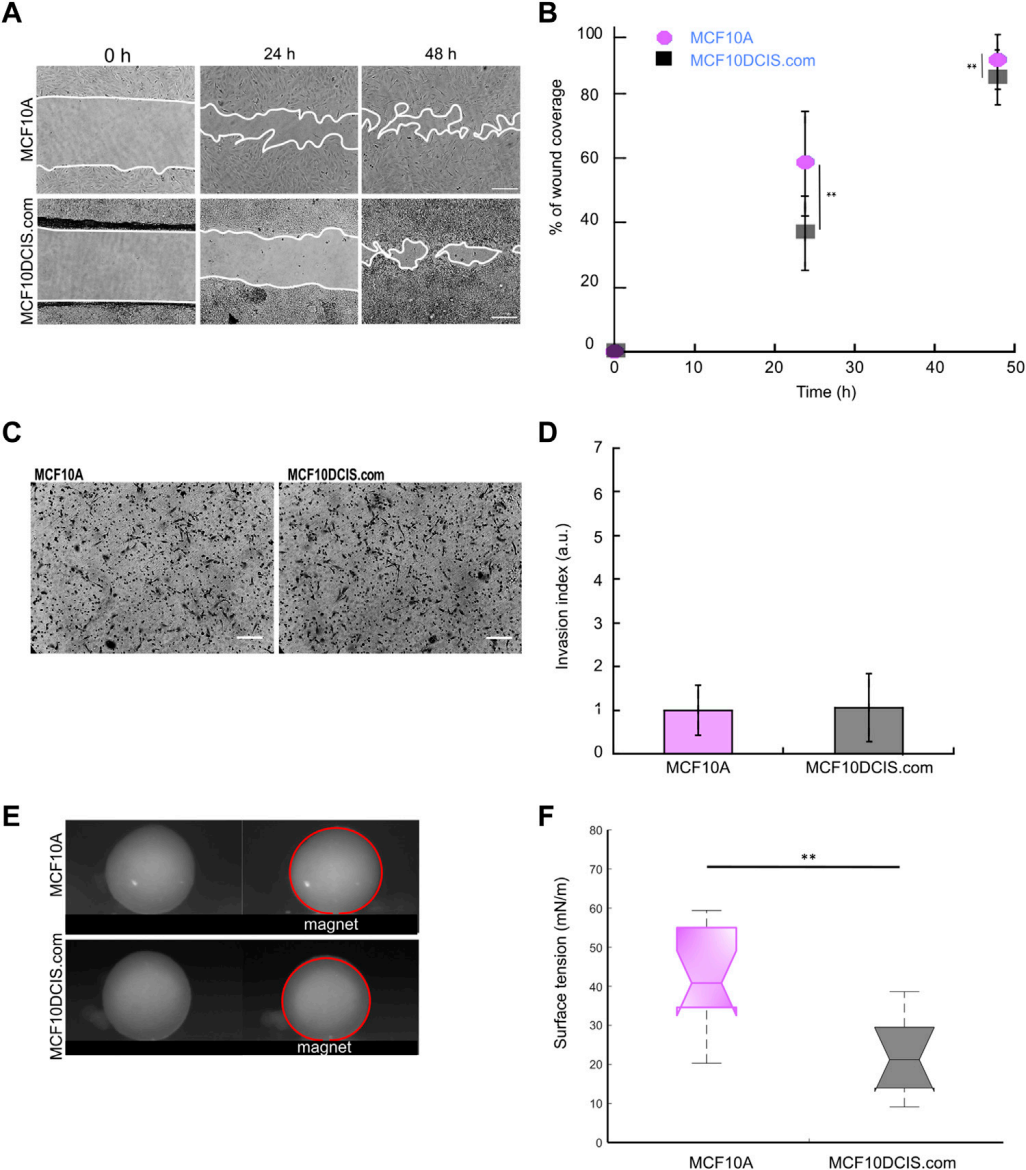
Behavior of the normal-like MCF10A cells compared to *in situ* carcinoma MCF10DCIS.com cells Three parameters are studied: **(A** and **B)** the migration over a scratch wound within 48 h, as shown in Figure 2, **(C** and **D)** the invasion through Matrigel using a Boyden chamber assay, as shown in Figure 3, and **(E** and **F)** the surface tension of multicellular aggregates using a magnetic tensiometer, as shown in Figure 5 (**p < 0.01).

MCF10A cells are considered a common normal-like breast cell model. These cells are derived from the benign proliferative breast tissue and are not tumorigenic (Soule et al., 1990). From them, genetic alterations have been created to study breast tumor progression. Here we studied one of its derivatives, the *in situ* carcinoma cells, MCF10DCIS.com, in which NME1 or NME2 were ablated. To find the potential of surface tension measurements in malignant transformation, we compared migration, invasion, and surface tension of normal-like MCF10A cells to carcinoma MCF10DCIS.com cells. Directional migration is enhanced in MCF10A cells compared to MCF10DCIS.com cells (Figure 6B). The migration mode in the 2 cell types differs as MCF10A cells are epithelial and migrate collectively, while MCF10DCIS.com cells migrate randomly with a more individual mode (Figure 6A). The invasion of the basement membrane is not modified by the malignancy state of the cells and is the same as the one measured for the NT MCF10DCIS.com control cells (Figures 6C,D). In sharp contrast, the surface tension of the multicellular aggregates from MCF10A cells is significantly higher than that of the multicellular aggregates from MCF10DCIS.com, 45 ± 18 mN/m vs. 21 ± 9 mN/m, respectively (Figures 6E,F). At the same time, the detachment force of the MCF10A cells has been reported to be in the 2.5 nN range (Pawlizak et al., 2015), while MCF10DCIS.com cells have a detachment force in the 2 nN range according to our data. Thus, surface tension also decreases during the transition from a normal state to a malignant cellular state.

## Discussion

We focus on a model of epithelial–mesenchymal transition induced by the loss of the metastasis suppressor NME1 in breast tumor cells. In this model, we first investigated the associated hallmarks of EMT by measuring cell–cell adhesion force, cell migration, and invasion. For this, we compared three different cell lines: ductal breast carcinoma *in situ* that are considered control tumor cells and two derivative cell lines obtained by inactivation of either NME1 or its closely related isoform NME2. AFM measurements show that loss of NME1 decreases cell–cell adhesion force, which corresponds well to the observed reduction of cell surface levels of E-cadherin upon NME1 inactivation (Huna et al., 2021). Previous functional aggregation and dispersion assays in the same cell line (Huna et al., 2021) already raised the possibility of modification of cell-cell adhesion but AFM measurements extend it to premature and early cell–cell links with a 60 s contact and fully quantify cell–cell adhesion force. The decrease of cell–cell adhesion force although moderate compared to the strong effects observed both in E-cadherin cell surface level and aggregate size after dispersion, suggests that quickly forming intercellular adhesions as well as the number of links is modified by NME1 inactivation.

Migration by mimicking potential diffusion properties of malignant cells and invasion constitute important read-outs for EMT (Friedl and Wolf, 2003). The loss of NME1 leads to increased 2D directional migration in a wound healing assay and 2D invasion through Matrigel, which mimics the basement membrane, in a Boyden chamber assay. By contrast, the absence of NME2, which is highly homologous to NME1, has no effect on cell–cell adhesion, cell migration, or invasion in the same tumor cells, indicating a highly specific function of NME1 in these EMT-associated biological processes. NME1 has been identified as the first metastasis suppressor, showing reduced expression in high melanoma metastatic cells and as a suppressor of breast, liver, and colon carcinoma metastasis through mechanisms that are not yet well-understood (Steeg et al., 1988; Boissan et al., 2005).

Several studies have demonstrated a fundamental role for NME1 in the promotion of endocytosis through interaction with dynamin. This endocytic function of NME1 could contribute to its activity towards the regulation of cell–cell adhesion, cell migration, and invasion during tumor progression. NME1 can promote the turnover of adherents junction components, including E-cadherin, through its endocytic controlling function, which is crucial for epithelial integrity (Palacios et al., 2002; Woolworth et al., 2009). In cooperation with dynamin, NME1 also contributes to the suppression of cell migration by promoting endocytosis of chemotactic receptors at the cell surface of migrating cells (Hsu et al., 2006). Indeed, NME1 facilitates the downregulation of activated chemotactic receptors *via* dynamin-mediated endocytosis, whereas the absence of NME1 increases the levels of cell surface receptors which results in oversensitized cells to chemotactic signals and elevated cell migration. Finally, NME1 promoted dynamin-mediated endocytosis of the transmembrane metalloproteinase MT1-MMP, known as a key player in tumor invasion, resulting in a strong reduction of surface MT1-MMP levels and a concomitant reduction of extracellular matrix degradation and invasion (Lodillinsky et al., 2021). Thus, promoting endocytosis is a major function of NME1 that limits EMT-related processes.

The physical and biological mechanisms regulating EMT and tumor progression have been determined in 2D *in vitro* assays, but the dimensionality of the environment is a key factor to understand tumorigenesis (Friedl et al., 2012). Two-dimensional cultures fail to recapitulate the three-dimensional structure of a tumor. Therefore, the translation of these properties from 2D to 3D is critical and has to be explored. In a purely cellular 3D environment, cell–cell adhesions are required for the cohesion of the cell assembly. Multicellular aggregates appear to be the most appropriate three-dimensional models to study mechanical properties (Gonzalez-Rodriguez et al., 2012; Giverso et al., 2019) and they represent an excellent model of the macroscopic behavior of tissues (Costa et al., 2016; Nikolaev et al., 2020; Ackermann et al., 2021). Being an intermediate stage between cell monolayers and biological tissues (Lin et al., 2008), 3D models recapitulate numerous biological processes while being more easily monitored and reproducible. We used magnetic techniques to rapidly form 1 mm spheroids for observation of the macroscopic properties of tissues (Mazuel et al., 2015). While NME1 inactivation impairs epithelial integrity in 2D culture, this effect is not conserved in 3D as the localization of E-cadherin is not impacted in spheroids. This decrease in impact of NME1 inactivation in 3D spheroids is also reflected in E-cadherin expression, as the lack of E-cadherin is less important in spheroids compared to NT-control cells. The 3D environment is based on cell–cell adhesion formation, thus increasing the expression of E-cadherin. Cell–cell interaction enhancement has already been observed in 3D culture environments in the context of tumor cell spheroids (Bissell et al., 2002; DesRochers et al., 2012; Kim et al., 2019). E-cadherin expression is controlled both by epigenetic and environmental factors during cancer progression, while the loss of E-cadherin has been proven to be reversible in breast cancer. E-cadherin expression has been restored in tumor spheroids *via* the demethylation of the E-cadherin promoter in a model of breast cancer (Graff et al., 2000). In contrast to E-cadherin, the level of N-cadherin is enhanced in these model tissues upon NME1 inactivation. Finally, the 3D environment recapitulates the overall effects of NME1 inactivation observed in 2D cultures and confirms the intermediate EMT state of NME1 ablated cells while reducing the difference with the NT-control cells.

**FIGURE 7:**
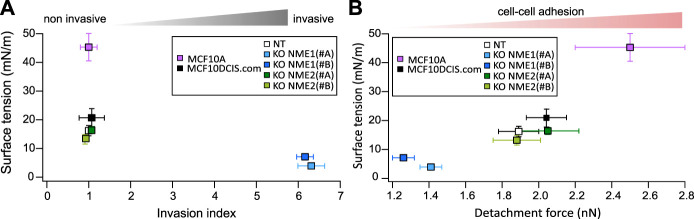
Surface tension comparison with invasion and adhesion. **(A)** The results obtained for the surface tension of aggregates from normal-like cells (MCF10A) and tumoral cells (MCF10DCIS.com and derivatives) are reported as a function of their invasion index. Means are indicated, and error bars show the SEM. **(B)** The results obtained for the surface tension of aggregates from normal-like cells and tumoral cells are reported in relation to their adhesion force measured by AFM after a 60 s contact. MCF10A adhesion force is extracted from Pawlizak et al. (2015). Means are indicated, and error bars show the SEM.

Material properties derived from soft matter concepts reveal as powerful tools to describe and predict the behavior of living tissues (Gonzalez-Rodriguez et al., 2012). Among them, surface tension depends mainly on adhesion and tension and is related to shape determination and maintenance. Our data show that surface tension is strongly decreased upon NME1 inactivation but not by NME2 inactivation. Surprisingly, this decrease is more important than the one measured by cell–cell adhesion determination, and it appears to be a translating difference observed in migration and invasion assays as highlighted in Figure 7. Surface tension is a complex macroscopic property related to effective adhesion, which considers intercellular adhesion as well as cell tension. This means that there may be some reinforcement due to the multiple impacts of NME1 both on adhesion and on cell stiffness. Importantly, epithelial cells have been shown to spontaneously convert to a mesenchymal migratory and invasive phenotype when plasma membrane tension was reduced, and that increasing plasma membrane tension was sufficient to suppress tumor migration, invasion, and metastasis (Tsujita et al., 2021). Remarkably, surface tension is measured in a 3D environment, and the effect of NME1 may actually be reinforced by the dimension change as cell–cell adhesion is at the heart of tissue modelling and shape maintenance. The mechanical properties of cells are regulated by their environment. While the influence of the extracellular matrix has been extensively studied (Levental et al., 2009; Stowers et al., 2019), the impact of cell organization and dimension is emerging as essential (Long Han et al., 2019).

Surface tension also decreases when comparing normal to tumoral state. MCF10A cells are a model of normal-like breast cells, whereas MCF10DCIS.com are *in situ* breast carcinoma cells. Adhesion is significantly reduced during this transformation (Pawlizak et al., 2015), and surface tension actually reflects these changes (Figure 7). Surface tension recapitulates the evolution of cell–cell adhesion upon malignant transformation and EMT thus extending the DAH upon these processes (Figure 7B). Differences in surface tension are larger. Indeed, while a 30% increase is measured on detachment force, surface tension is actually doubled. There are two main reasons for this strengthening. First, the 3D structure strongly implicates cell–cell adhesions and may enhance adhesion changes. Second, while surface tension predominantly depends upon cell–cell adhesion, it is also affected by other biomechanical properties. The greater sensitivity of surface tension may actually reveal this multi contribution.

In addition, invasion measurements appear to be highly sensitive to EMT modifications but fail to distinguish between cells during malignant transformation (Figure 7A). Surface tension thus appears to be a full range indicator of biophysical modifications both in malignant transformation and EMT.

While individual cell stiffness has been identified as a potential biomarker of metastatic potential (Guck et al., 2005; Xu et al., 2012), surface tension arises as an indicator of malignant transformation and tumor aggressiveness that can be measured on tissue-like structures closer to the actual tumor environment than cells grown in 2D culture. This property appears to be highly sensitive to any changes in adhesion, while also being easier to measure. Abnormal cell–cell adhesion, as well as enhanced migration and invasion, stand as three major hallmarks of tumor aggressiveness. They clearly indicate tumor progression upon hybrid states encountered in EMT. Decreased surface tension appears to be an appealing fourth one. Indeed, it is highly correlated with the three major hallmarks of tumor progression in our breast tumor model. Moreover, surface tension acts as a read-out of malignant transformation (from normal to tumoral state) and as a read-out of tumor aggressiveness (from tumoral non-invasive to tumoral invasive state) in breast tumor models.

## Conclusion

These results demonstrate that surface tension through its multiparameter dependence reflects cell organization, mechanics, and adhesion and can serve as a sensitive indicator of the state of the cells undergoing EMT as well as through the transformation from a normal state to a malignant state. Important changes in surface tension are detectable in response to subtle phenotype changes. Surface tension proved to be highly sensitive to any changes in adhesion properties at the single-cell level while being measured at the more biomimetic scale of model tissue. We investigated the sensitivity of the magnetic tensiometer across the EMT using a model of EMT induced by the loss of NME1. NME1, by acting on cell–cell interactions through E-cadherin turnover, participates in the maintenance of tissue integrity and shape. Thus, surface tension can be considered a signature of tumor aggressiveness during the EMT. This new biophysical tool appears crucial in the investigation of metastatic potential in tridimensional environments.

## Data Availability

The raw data supporting the conclusion of this article will be made available by the authors, without undue reservation.

## Supplementary material

Supplementary Figure S1 Ablation of NME1 or NME2 expression assessed by Western blot analysis. MCF10DCIS.com human breast carcinoma cells were treated with two independent guide RNAs specific for the NME1 gene, NME1 (#A) and NME1 (#B), and two for the NME2 gene, NME2 (#A) and NME2 (#B). Cells without guides are NT control cells.

Supplementary Figure S2 Metabolic activity for unlabeled (CTL) and labeled cells (with NP) 2 h (D0) and 1 day (D1) after nanoparticle incorporation. To assess metabolic activity, the metabolic test Alamar Blue was used. Fluorescence was measured at *λ*_*exc*_ = 570 nm and *λ*_*em*_ = 585 nm. Values are interpreted relative to control values (unlabeled cells in complete medium, CTL) obtained under similar conditions. No influence of nanoparticle incorporation was observed on the metabolic activity at D0 or D1.

Supplementary Figure S3 Lack of impact of nanoparticles on cell–cell interaction, cell migration, and invasion. **(A)** Boxplots of detachment forces from NT control cells either labeled or not by nanoparticles (in average *n* = 45 cells were tested per condition). **(B)** Top: Representative light microscopy images of the wound healing assay of MCF10DCIS.com NT control cells either labeled (bottom) or not (top) with nanoparticles. Time 0 represents confluent monolayer wounds at 0 h and wounds were monitored until 48 h after scratching the monolayer. Images are representative of three independent biological replicates. Scale bar: 100 μm. Bottom: Quantification of the area of migration over time shown in the top part. Data show means ± SD of three independent biological replicates imaged. **(C)** Left: Representative light microscopy images of Matrigel invasion assay of MCF10DCIS.com NT control cells either treated or not with nanoparticles after 24 h. (Scale bar: 100 μm) Right: Quantitative analysis of the invasion assay presented at the left. The data show the means ± SEM of three independent biological replicates imaged and are expressed as an invasion index defined as the ratio of the number of invading magnetically labeled cells to the number of invading unlabeled cells. By definition, the invasion index of unlabeled cells is 1.

Supplementary Figure S4 Cell surface cadherin expression is reduced in spheroids of NME1-ablated cells. **(A)** Top left: image of a spheroid of 1 mm diameter of NT control cells. The observation plane is indicated in white. Top right: image obtained at 50 μm depth penetration. The position of the zoom is indicated by the white square. **(B)** Immunofluorescence confocal images of multicellular spheroids from MCF10DCIS.com cells in which NME1 or NME2 was inactivated taken at 50** **μm from the top of the aggregate. Pan-cadherin antibody labeling both N-cadherin and E-cadherin is used (green). The same conditions in terms of illumination and recording are applied. Images of nuclei labeled (blue) are added. (Scale bars: 20 μm) **(C)** Quantitative analysis of the mean intensity of the pan-cadherin labeling renormalized to the NT control cells. Means and standard deviations are represented. *N* = 4 aggregates were imaged for each condition. * *p* < 0.05, ** *p* < 0.01.
