# Supplementary figures and images for: Surface tension of model tissues during malignant transformation and epithelial–mesenchymal transition
Source: Front Cell Dev Biol. 2022 Aug 30;10:926322. doi: 10.3389/fcell.2022.926322 (PMC9468677; doi:10.3389/fcell.2022.926322)

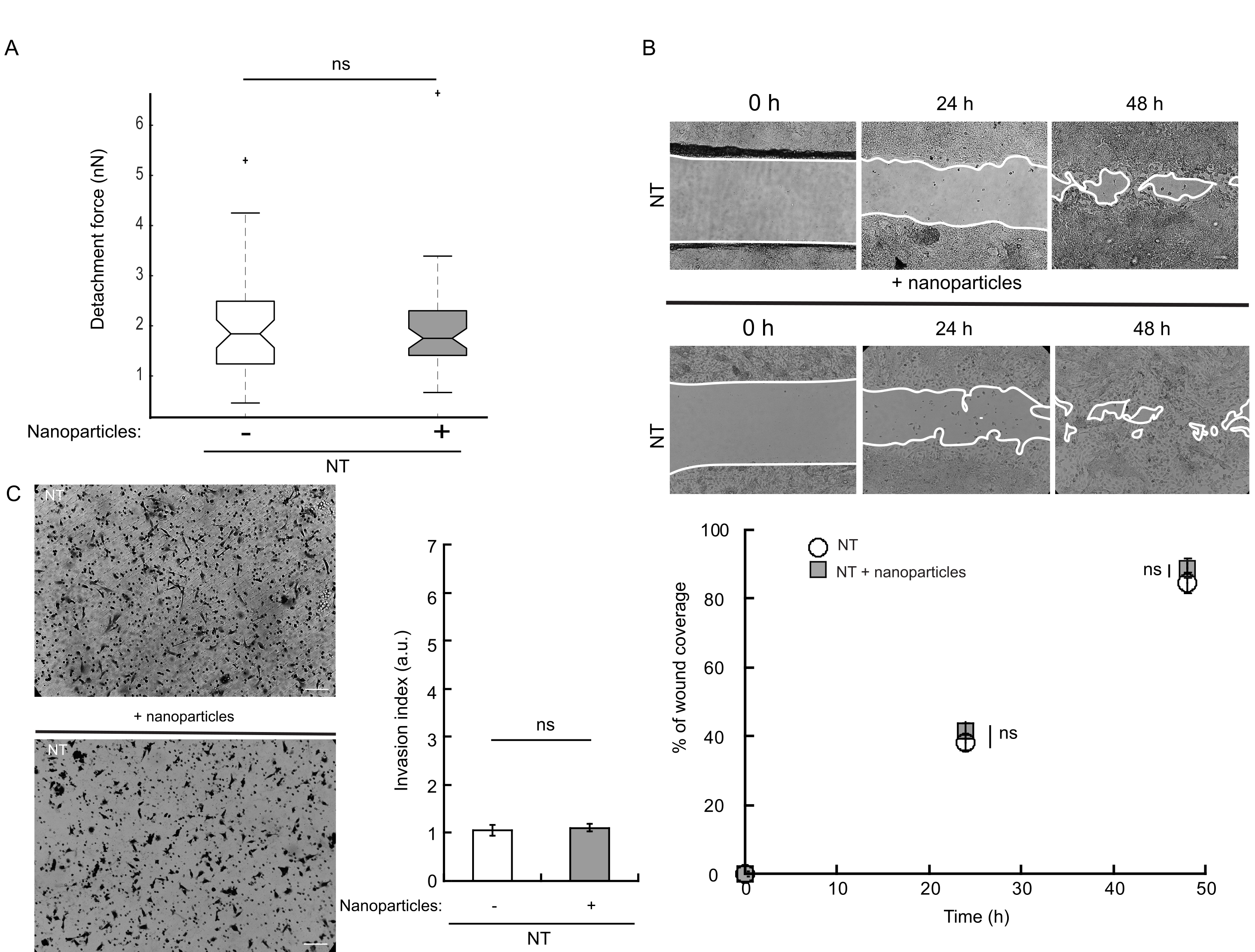

Supplement: Supplementary file 1 [file Image3.TIF]

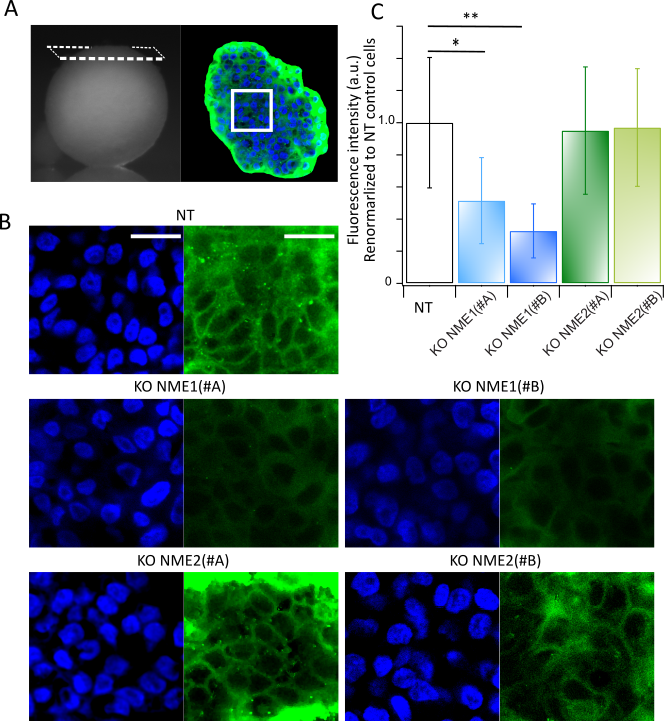

Supplement: Supplementary file 2 [file Image4.TIF]

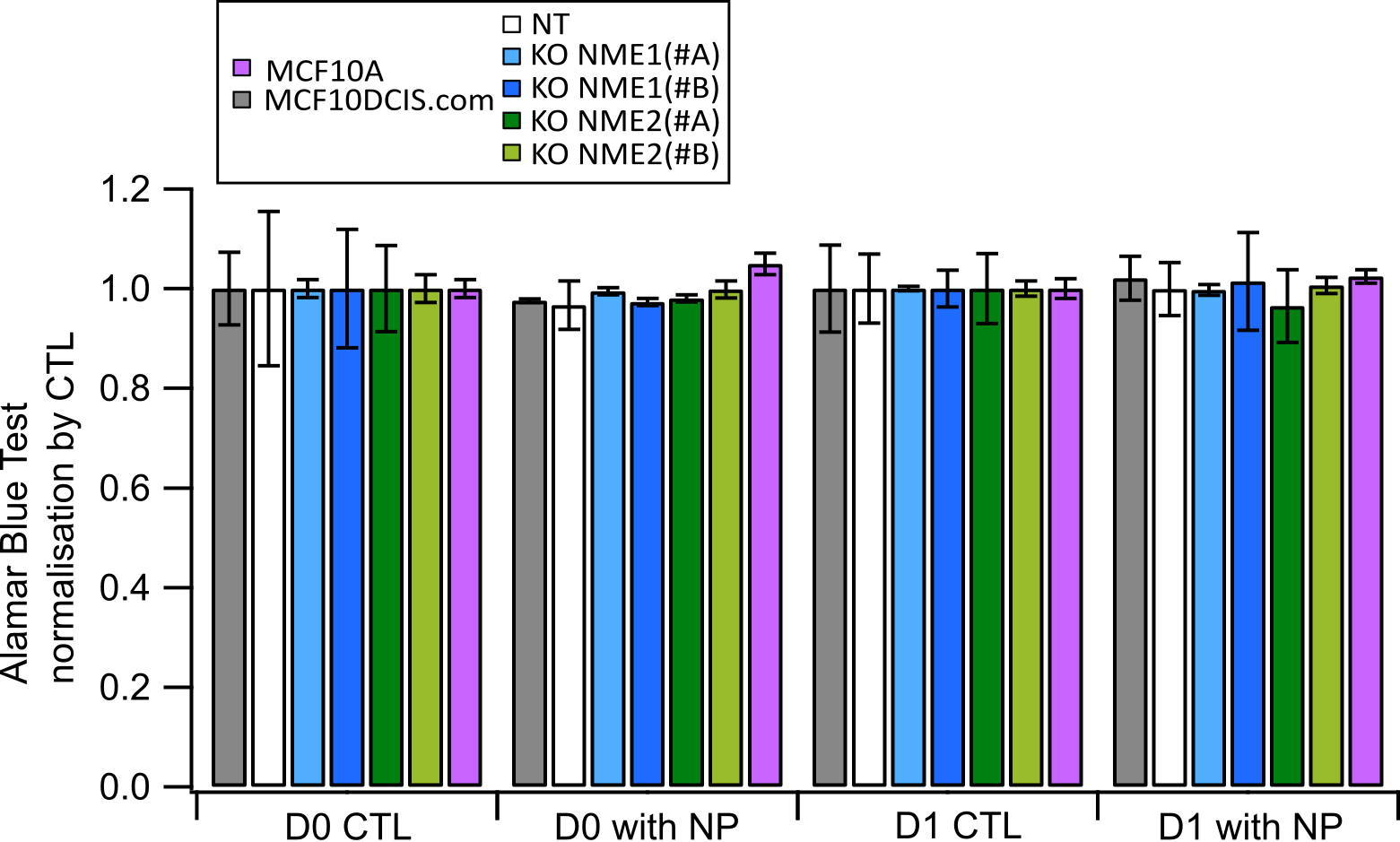

Supplement: Supplementary file 3 [file Image2.TIF]

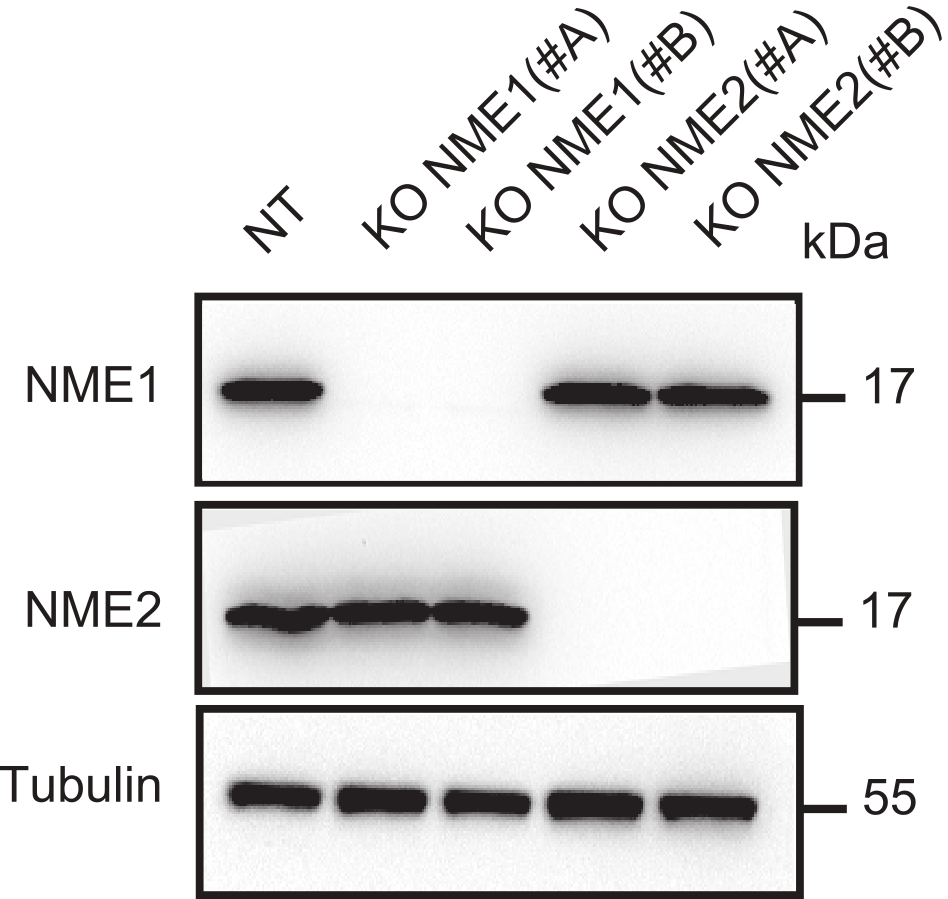

Supplement: Supplementary file 4 [file Image1.TIF]
